# Effects of triticale silage substitution for corn silage on growth performance, serum biomarkers, rumen fermentation, and rumen bacterial community of Pingliang Red Cattle

**DOI:** 10.1186/s12866-026-05245-8

**Published:** 2026-06-15

**Authors:** Weiqiang Wang, Wenhua Du, Fangyuan Zhao, Qian Song, Junsheng He

**Affiliations:** 1 College of Grassland Science, Gansu Agricultural University, Key Laboratory of Grassland Ecosystem of Education Ministry, Pratacultural Engineering Laboratory of Gansu Province, Sino-U.S. Centers for Grazingland Ecosystem Sustainability, Lanzhou, 730070 China; 2 Institute of Grass and Livestock, Pingliang Academy of Agricultural Sciences, Pingliang, 744399 China

**Keywords:** Growth performance, Rumen fermentation, Rumen microorganism, Serum marker, Triticale silage

## Abstract

**Background:**

In the Loess Plateau region, corn silage production is constrained by water scarcity and rising costs. Triticale silage offers a promising alternative, yet its impact on the performance, metabolism, and rumen microbiota of Pingliang Red Cattle (PRC) remains poorly understood. This study therefore evaluated the effects of substituting corn silage with triticale silage on growth performance, pancreatic enzyme activity, serum biochemistry, rumen fermentation, and the rumen microbial community in PRC, and applied the TOPSIS method to determine the optimal substitution ratio.

**Methods:**

Thirty clinically healthy 18-month-old PRC bulls with good appetite (initial body weight, 400 ± 50 kg) were selected and assigned to a completely randomized design. Animals were randomly allocated to five groups (*n* = 6 per group), including a control group fed 100% corn silage (T0) and four treatment groups in which triticale silage substituted corn silage at 30% (T1), 50% (T2), 70% (T3), and 100% (T4), respectively. Except for the type and proportion of silage, all other dietary components were kept constant. The experiment consisted of a 14-day adaptation period followed by a 90-day feeding trial.

**Results:**

With increasing substitution level, trypsin (TS) and chymotrypsin (CT) activities, and serum alkaline phosphatase (ALP) activity were significantly elevated (*P* < 0.01); urinary urea nitrogen (UUN) concentration tended to decrease; rumen ammonia nitrogen (NH_3_-N) concentration was significantly reduced (*P* < 0.01); total volatile fatty acids (TVFA) and microbial crude protein (MCP) contents increased; and the relative abundances of core functional taxa (*Firmicutes*, *Bacteroidota*, *Prevotella*) also rose. However, dry mater intake (DMI) and average daily gain (ADG) decreased significantly (*P* < 0.05) with increasing substitution ratio.

**Conclusions:**

The TOPSIS analysis, integrating all evaluated parameters (growth performance, serum biomarkers, rumen fermentation, and microbial community), identified the 30% substitution ratio as optimal. At this level, production performance remained comparable to the T0, while protein digestion, metabolic balance, rumen fermentation, and microbial community structure were improved, achieving integrated benefits across digestive, metabolic, and microbial dimensions, thereby directly addressing the study’s objective to determine the optimal substitution ratio.

**Graphical abstract:**

**Figure:**
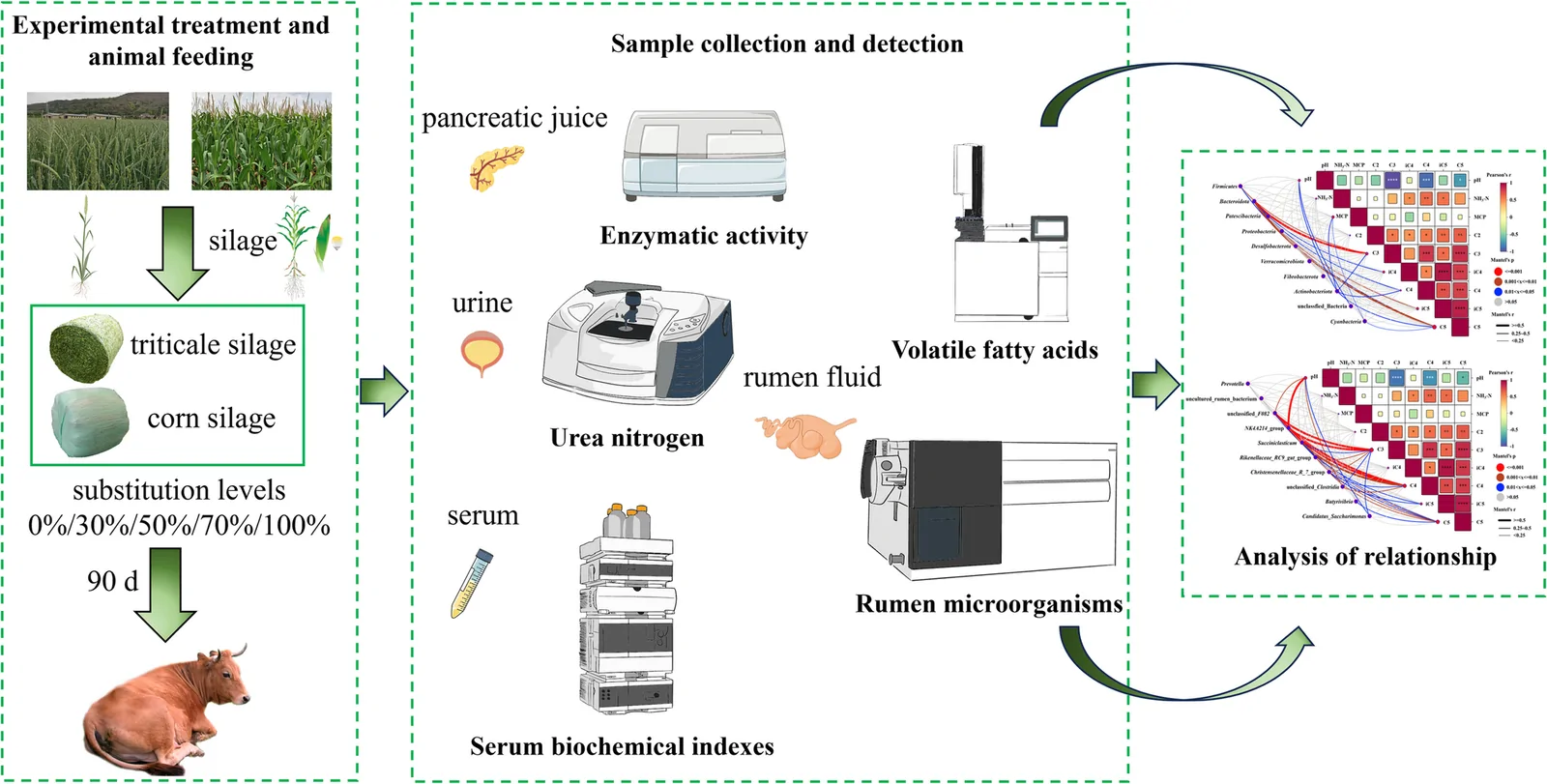

## Introduction

With the global livestock industry shifting toward high efficiency and sustainability, optimizing the allocation of feed resources has become crucial for ensuring animal productivity, improving breeding benefits, enhancing animal health, and reducing environmental burdens [1]. Among the most essential roughages in ruminant diets, silage offers key advantages such as effective nutrient preservation, good palatability, stable fermentation performance, and year-round supply. It serves an irreplaceable function in maintaining stable rumen fermentation, enhancing nutrient utilization efficiency, and promoting the healthy growth of beef cattle [2]. In temperate, arid, and semi-arid regions, silage helps mitigate seasonal forage shortages and stabilizes feed supply, making it increasingly strategic for large-scale beef production. In China, corn silage remains a cornerstone of beef cattle diets owing to its high yield, energy density, and reliable fermentation [3, 4]. However, corn cultivation demands substantial water and fertilizer inputs. In arid and semi-arid regions like the Loess Plateau, insufficient precipitation and limited irrigation result in unstable corn silage yield and quality [5]. Moreover, rising feed costs and growing roughage demand mean a feeding system dependent solely on corn silage can no longer satisfy the development requirements of the local beef cattle industry. Consequently, developing local, low-input, highly adaptable alternative silage resources with favorable nutritional value is critical for mitigating forage shortages, reducing feeding costs, and ensuring stable production.

PRC is an important local beef cattle breed in the Loess Plateau region, renowned for its tender meat, roughage tolerance, and strong stress resistance [6]. Nevertheless, the natural grassland resources in its main production areas are limited, and green forage has a short growth cycle, resulting in particularly tight forage supply during winter and spring. With the continuous expansion of breeding scales, the contradiction between supply and demand of corn silage has become more prominent, and forage shortage has emerged as the primary bottleneck restricting the production potential and industrial development of PRC [7]. Therefore, screening roughage that is suitable for local cultivation, has a reasonable nutritional structure, and can partially substitute corn silage is a key approach to promoting the sustainable development of the PRC industry.

Triticale (×*Triticosecale* Wittmack), a dual-purpose grain and forage crop, has gained rapid promotion in major corn-producing regions and forage-deficient areas of northern China in recent years [8]. It exhibits remarkable regional adaptability, particularly in the main production areas of PRC, with advantages including cold tolerance, drought resistance, low soil requirements, controllable planting costs, and stable yields. Furthermore, triticale silage is characterized by high crude protein (CP) content, good crude fiber digestibility, and suitable fermentation quality, which endows it with the potential to serve as an alternative to corn silage. This substitution may influence the substrate availability in the rumen, thereby potentially modulating the ruminal microbial community structure and fermentation patterns, which are critical determinants of nutrient harvest and animal performance. This provides an effective option for constructing a robust, economical, and sustainable dietary system for beef cattle [9].

Existing studies have demonstrated that substituting triticale silage for corn silage does not significantly alter the hematological parameters of dairy cows, nor does it compromise animal health status, suggesting that triticale silage can be safely incorporated into dairy cow diets [10]. Furthermore, although substituting barley silage with triticale silage decreases ruminal pH and affects feed intake, it does not significantly influence animal growth performance and carcass quality. These findings suggest that barley silage may be substituted with triticale silage in the finishing diet [11]. In addition, *Pediococcus pentosaceus* (TC48) and *Lactobacillus brevis* (TC50), isolated from fermented triticale silage, can significantly enhance silage quality (e.g., high antibacterial activity, probiotic potential), thereby optimize triticale silage fermentation and improve its nutritional value [12]. However, the interactions among the rumen microbiota, dietary structure, and host physiology, as well as the cascading responses triggered by such feed substitution, remain poorly understood. Most existing studies have focused predominantly on dairy cattle, whereas systematic research on local indigenous beef breeds such as PRC remains scarce. Moreover, most investigations have been limited to single indicators including growth performance, feed fermentation, or rumen fermentation, and have not established a systematic linkage among dietary substitution, rumen microbiota, host metabolism, and phenotypic traits. Specifically, the intrinsic mechanism by which triticale silage substitution reshapes rumen microbial community structure in terms of diversity, composition, and functional groups, and further modulates rumen fermentation profiles and host metabolic status, remains unclear. Meanwhile, studies that comprehensively evaluate the effects of such substitution on growth performance, serum biomarkers, rumen fermentation, and microbial community structure are still lacking.

Accordingly, the present study was conducted using PRC, a representative local beef breed, and focused on triticale silage with strong regional adaptability and substitution potential. A comprehensive evaluation was performed covering growth performance, serum biochemical indices, pancreatic enzyme activities, rumen fermentation characteristics, and rumen bacterial community structure. Importantly, we integrated dietary substitution, rumen microecology, host metabolism, and production phenotypes into a unified analytical framework. Compared with previous studies that focused on dairy cattle or relied on single‑indicator analysis, the present study expands both the research scope and methodological system, thereby filling critical knowledge gaps in this field.

By systematically elucidating the effects of substituting corn silage with triticale silage on PRC’s growth performance, serum biochemical indices, ruminal fermentation characteristics, and ruminal bacterial community structure, this study provides theoretical support for the precise optimization of triticale silage substitution strategies in ruminant diets.

## Materials and methods

### Experimental site

The study site (Shouyan Animal Husbandry Co., Ltd., Jingchuan County, Pingliang City, Gansu Province, China) is located at an altitude of 1,308.0 m, receives 555.0 mm precipitation annually, and has an average annual temperature of 10.3 °C. It has a typical temperate semi-humid continental climate.

### Animals, experimental design, feeding management, and sampling

The triticale variety used in this experiment was ‘Gannong No. 2’, cultivated by the College of Grassland Science, Gansu Agricultural University; the corn variety was ‘Yu Silage 23’, provided by Shouyan Animal Husbandry Co., Ltd., Jingchuan County, Pingliang City. The triticale and corn were harvested at the mid-milk stage (May 15, 2021) and early dough stage (1/2 milk line) (September 20, 2021), respectively. After harvesting, both forages were chopped to a length of approximately 2 cm using a silage harvester, then compacted and baled with a baler (bale density controlled at 550–650 kg/m^3^). each silage bale (weighing approximately 200 kg) was hermetically sealed with multiple layers of silage stretch film (specifications: 25 μm × 250 mm; manufacturer: Baoruita (Cangzhou) Packaging Co., Ltd.). the sealed bales were placed in a cool, shaded environment (temperature: 20–28°C) for natural fermentation, after 60 days of natural fermentation (by which time the triticale silage had been stored for approximately 4 months), the corn silage was opened simultaneously with the triticale silage. First, their sensory qualities (color, odor, and texture) were evaluated according to relevant standards. Subsequently, samples were taken to determine fermentation parameters (pH, NH_3_-N, organic acids) and nutritional composition, confirming that both silages had reached a stable fermentation state. To eliminate the confounding effect of moisture differences between the raw materials, the experimental diets were precisely formulated based on the measured Dry Matter (DM) content. Furthermore, by optimizing the diet structure, the metabolizable energy (ME) level was kept balanced across all groups with no significant differences (*P* > 0.05), thereby effectively avoiding any interference from variations in energy levels. The silages processed as above were then used for the subsequent diet preparation.

The study used thirty non-castrated PRC bulls provided by Shouyan Animal Husbandry Co., Ltd. in Jingchuan County, Pingliang City. All animals were quarantined and held valid *Animal Quarantine Certificates* prior to the experiment. They had an initial body weight of 400 ± 50 kg, with similar age (18 months old), exhibited good appetite, and were clinically healthy. A completely randomized design was adopted, with the cattle randomly divided into five groups of six individuals each, and were housed in individual stalls to ensure independent replication. The environmental temperature was maintained at approximately 25℃ with natural light. The cattle were fed a total mixed ration (TMR) at 08:00 and 17:00 daily, with 5%-10% orts to ensure ad libitum intake. Feed offered and orts were recorded daily to calculate dry matter intake (DMI). Feeding behavior, rumination, mental status, and fecal consistency were continuously observed, and the stalls, feed troughs, and water troughs were regularly cleaned and disinfected. One-way analysis of variance (ANOVA) showed that there were no significant differences in initial body weight and age among the groups (*P* > 0.05). The group T0 was fed a basal diet containing 100% corn silage, while groups T1, T2, T3 and T4 were fed diets with 30%, 50%, 70% and 100% triticale silage substituting corn silage, respectively. Owing to the nutritional differences between the two silage types, the proportions of corn kernels, soybean meal, and other concentrate ingredients were adjusted based on the measured DM content to keep CP levels, the roughage-to-concentrate ratio, and ME as consistent as possible across treatment groups. All diets were formulated according to the nutritional standards for beef cattle of the National Research Council (NRC, 2016) [13], and a commercial premix (NBD-512) was used to meet the mineral and vitamin requirements of finishing cattle. The experiment included a 14-day pre-trial period starting on March 8, 2022 and a 90-day formal trial period that concluded on June 25, 2022. The pre-trial period was used for diet transition and metabolic adaptation. The composition and nutritional levels of the diets are shown in Table 1.

**Table 1:** Composition and nutrient composition of diets for each experimental treatment (DM level, %)

Items ^a^		Treatment ^b^				
		T0	T1	T2	T3	T4
Roughage	Ingredient composition (% DM)					
	Corn silage	64.00	44.80	32.00	19.20	—
	Triticale silage	—	19.20	32.00	44.80	64.00
	Wheat straw	7.80	7.80	7.80	7.80	7.80
Concentrate	Corn kernels	20.10	21.65	22.30	23.00	23.50
	Soybean meal	3.90	2.80	1.70	1.00	0.50
	Bran	1.50	1.50	1.50	1.50	1.50
	NaCl	0.30	0.30	0.30	0.30	0.30
	Premix	1.20	1.20	1.20	1.20	1.20
	NaHCO_3_	1.00	1.00	1.00	1.00	1.00
	Urea	0.20	0.20	0.20	0.20	0.20
	Total	100.00	100.00	100.00	100.00	100.00
	Nutrition level					
Nutritional composition	Chemical composition (% DM)					
	CP	15.17	14.63	15.26	15.43	15.58
	ST	30.50	28.90	27.30	25.70	24.10
	ADF	22.00	22.70	23.40	24.10	24.90
	NDF	39.50	40.30	41.10	41.90	42.70
	Ca	0.59	0.57	0.58	0.61	0.63
	P	0.54	0.52	0.52	0.54	0.55
	TA	0.12	0.14	0.15	0.16	0.17
	Fatty acids composition (% of total fatty acids)					
	C16:0	0.47	0.43	0.39	0.35	0.31
	C18:0	0.07	0.06	0.05	0.04	0.03
	C18:1	0.73	0.56	0.42	0.27	0.08
	C18:2	1.35	1.05	0.87	0.60	0.30
	C18:3	0.03	0.26	0.41	0.61	0.86
	UFA	2.18	1.93	1.75	1.52	1.27
	Organic acid (% DM)					
	La	1.59	3.02	4.38	3.49	4.76
	C2	1.54	2.31	3.07	3.02	2.61
	C3	0.24	0.40	0.58	0.52	0.25
	ME (MJ/kg) ^c^	8.28	8.22	8.10	8.03	7.92

During the operation of substituting corn silage with triticale silage, in accordance with the preset substitution ratios of each experimental group, the DM contents of triticale silage and corn silage within the total DM of the daily ration are first determined. These DM contents are then converted into the actual weights of the silage feeds to be used, based on which the daily rations for each experimental group are formulated

The additive remix provided the following per kilogram of the diet: VA: 20,000 ~ 150,000 IU, VD: 50,000 ~ 80,000 IU, VE > 500 mg, Ca: 8%~18%, P: 2.0%~5.5%, NaCl: 10%~20%, Fe: 0.5 ~ 1.5 g, Cu: 0.1 ~ 0.5 g, Zn: 0.5 ~ 2.0 g, Mn: 0.4 ~ 2.0 g, Se: 5 ~ 10 mg, I: 10 ~ 15 mg, Co: 4 ~ 10 mg

^a^CP, crude protein; ST, starch; ADF, acid detergent fiber; NDF, neutral detergent fiber; Ca, calcium; P, phosphorus; TA, tannins; UFA, Unsaturated fatty acid; La, lactic acid; C2, acetic acid; C3, propionic acid; ME, metabolizable energy

^b^T0, 100% corn silage, T1, 30% triticale silage substitution, T2, 50% triticale silage substitution, T3, 70% triticale silage substitution, T4, 100% triticale silage

^c^The metabolic energy (ME) was estimated from ADF content of the diets according to the empirical equation: ME (MJ/kg DM) = − 0.195 × ADF (%) + 15.32, where ADF is expressed on a DM basis. All other chemical composition values were determined by laboratory analysis

After the experiment, the cattle were moved to the slaughter area 12 h before slaughter and stopped feeding. Water was stopped 3 h before slaughter. The eviction process is kept quiet, and any beating or scaring was strictly prohibited to minimize stress and physical exertion. A single-pole manual contact electric shock device (DYX280, Zhucheng Zhengfeng Machinery Factory) was used for slaughter-induced fainting. During operation, the electroshocker electrodes were firmly attached to the forehead of the cow (the intersection of the wires connecting the ears and the wires connecting the eyes), and the shock parameters were set to a voltage of 200 V and a current of 1.2 A for 3–5 s to ensure immediate unconsciousness. The unconsciousness effect was confirmed by the animal’s instantaneous collapse and loss of consciousness (e.g., loss of corneal reflex), followed by immediate exsanguination and slaughter. This procedure followed the requirements of the *Guidelines for the Ethical Review of Laboratory Animal Welfare* to ensure animal welfare and reduce suffering.

### Growth performance

The ADG was calculated with reference to the method described by Saleem et al. [14]. All experimental PRC were weighed in a fasted state (12 h of feed deprivation and 2 h of water deprivation) on the first day of the trial, with the initial body weight (*BW*_*initial*_) and final body weight (*BW*_*final*_) recorded. Meanwhile, the total duration of the trial (*T*, unit: days) was accurately documented. ADG represents the average daily weight gain of the cattle over the trial period, and its formula is: ADG (kg/day) = (*BW*_*final*_ − *BW*_*initial*_) / *T*.

The TFI was calculated following the method of Wang et al. [15], the daily feed offered (*Feed*_*offered*_, unit: kg) for each individual (or group) of experimental cattle was recorded at a fixed time every day during the trial period. Before feeding on the following day, the refused feed (*Feed*_*refused*_, unit: kg) was collected and weighed to calculate the daily net feed intake (Feed offered − Feed refused). TFI is the sum of all daily net feed intake values over the trial period, and its formula is: TFI (kg) = $\:\sum\:_{\mathrm{i}{=}{1}}^{\mathrm{T}}(Feed_{offered},\:i\:-\:Feed_{refused},\:i)$ (where *i* represents the *i*-th day of the experiment).

The determination of TG relies on the initial and final body weight data recorded during the measurement of ADG, representing the total body weight change of experimental cattle over the trial period. Its formula is: TG (kg) = *BW*_*final*_ − *BW*_*initial*_.

The DMI represents the average daily net feed intake during the trial period, and its formula is: DMI (kg/d) = TFI / *T*.

The FCR represents the amount of feed required to produce 1 kg of body weight gain, and its formula is: FCR = TFI / TG.

### Collection and analysis of pancreatic juice and urine

Prior to the conclusion of the trial, a sterile silicone catheter (outer diameter: 2–3 mm) was inserted to a depth of 1–2 cm at the pancreatic duct orifice and secured to the duodenal wall. During sampling, a drainage bag was aseptically connected to the catheter, with naturally secreted pancreatic juice draining into the bag via the catheter without manual intervention. Pancreatic juice samples were collected hourly; 10% of each sample (approximately 3–5 mL) was immediately measured for volume and transferred to a pre-chilled centrifuge tube supplemented with a protease inhibitor. Following centrifugation at 1,050 ×*g* for 10 min at 4 °C (H2050R, Xiangyi Laboratory Instrument Development Co., Ltd., Hunan, China), the supernatant was harvested, snap-frozen in liquid nitrogen, and stored at − 80 °C until subsequent analysis. The remaining pancreatic juice was allowed to flow back into the intestine spontaneously via the catheter (with no manual intervention) to preserve normal digestive function. Sterile conditions were rigorously maintained throughout the entire sampling procedure.

Over three consecutive days prior to the trial conclusion, 24-h composite urine samples were collected from experimental animals using specialized urine collection bags before morning feeding. Prior to collection, the vulva was disinfected with a 0.1% benzalkonium bromide solution and rinsed with sterile saline, after which urine was collected during natural micturition. Sodium azide (NaN_3_; 0.02% final concentration, *w*/*v*) was added to inhibit microbial proliferation. Following a 3 min standing period, solid contaminants were removed by filtration through four layers of sterilized gauze. The filtrate was transferred to sterile centrifuge tubes and centrifuged at 1050 ×*g* for 10 min at 4 °C (H2050R, Xiangyi Laboratory Instrument Development Co., Ltd., Hunan, China). The resulting supernatant was aliquoted into 5-mL sterile cryopreservation tubes, snap-frozen in liquid nitrogen, and stored at − 80 °C until subsequent analysis.

The activities of CT, TS, and lipase (LS) in pancreatic juice were quantified via colorimetric assays. Additionally, the UUN concentration in urine was determined using a urease-based colorimetric method. All photometric analyses were conducted using a microplate reader (BioTek Epoch 2 Instruments, Inc., USA). Commercial kits acquired from the Nanjing Jiancheng Institute of Bioengineering were utilized for the determination of all analytes.

### Collection and analysis of serum

On the last day of the trial period, 75% medical alcohol and iodophor were used to disinfect the blood collection site (from inside to outside) on the neck of fasted cattle before morning feeding. A sterile vacuum blood collection tube was used to collect 5 mL blood following jugular vein puncture. The blood collection tube was tilted (30°–45° angle) to promote the natural inflow of blood. Blood samples were left standing at room temperature for 30 min and then centrifuged at 1,050 ×*g* for 10 min at 4 °C (H2050R, Xiangyi Laboratory Instrument Development Co., Ltd., Hunan, China). The upper clear serum was carefully collected using a sterile pipette to avoid touching the precipitated blood cells and then sub-packed into a sterile cryopreservation tube, immediately frozen in liquid nitrogen, and stored at − 80 °C until analyzed.

ALT, AST, albumin (ALB), total protein (TP), total bilirubin (TBIL), direct bilirubin (DBIL), ALP, CREA, uric acid (UA), glucose (GLU), triglyceride (TRG), cholesterol (CHO), calcium (Ca), phosphorus (P), SOD, MDA, and GSH-Px activities or concentrations in serum samples (i.e., serum biomarkers) were determined by Wuhan Servicebio Technology Co., Ltd.

### Collection and analysis of ruminal fluid

Rumen fluid was collected on three consecutive days at the end of the trial, 3 h after morning feeding. After collection, rumen fluid samples were immediately filtered through four layers of cheesecloth and then processed according to the following procedures for different analyses. Approximately 5 mL of the filtered rumen fluid was transferred into a separate sterile sample tube, and the pH value was measured immediately using a properly calibrated pH meter. Another 5 mL aliquot of the filtered rumen fluid was acidified by the addition of 0.5 mL of 50% H_2_SO_4_ solution (final concentration 1%), gently mixed by inversion, and then centrifuged at 12,000 × *g* for 15 min at 4 °C (H2050R, Hunan Xiangyi Laboratory Instrument Development Co., Ltd., China). After centrifugation, the supernatant was carefully collected, transferred into pre-chilled sterile cryotubes, immediately snap-frozen in liquid nitrogen, and subsequently stored at − 80 °C until analysis for TVFA, NH_3_-N, and other fermentation parameters.

### Analysis of fermentation parameters and volatile fatty acids

Prior to pH determination, the pH meter (PB-10, Sartorius Group, Germany) was calibrated using pH 4.01 and 6.86 standard buffers. The probe was rinsed with distilled water, blotted dry with filter paper, and sequentially immersed in each buffer. After stabilization, calibration was confirmed in accordance with the manufacturer’s protocol. Fully oscillated rumen fluid was placed in a sterile tube and the acidity meter probe was inserted into the rumen fluid. After the reading stabilized, the value was recorded. Each treatment was repeated six times. After each sample was measured, the probe was washed with distilled water and dried with filter paper before analyzing the next sample. Additionally, the surrounding environment and temperature changes were considered.

The microbial crude protein (MCP) content was determined using an optimized version of the method described by Miguel et al. [16]. Briefly, 10 mL filtered rumen fluid was mixed with 10 mL pre-cooled 10% trichloroacetic acid solution. The mixture was placed in an ice bath for 30 min to fully precipitate proteins, transferred to a 50 mL centrifuge tube, and centrifuged at 17,000 ×*g* for 15 min at 4 °C (H2050R, Xiangyi Laboratory Instrument Development Co., Ltd., Hunan, China). After carefully discarding the supernatant, 10 mL acetone was added to the precipitate (acetone maintained below 4 °C), which was resuspended by vortexing or gently stirring and then centrifuged again at 17,000 ×*g* for 10 min at 4 °C. The supernatant was discarded and the precipitate was washed with acetone 2–3 times until the supernatant was colorless and transparent to remove impurities (e.g., lipids). The washed precipitate was dried at room temperature and resuspended in 1 mL phosphate buffered saline. A 200 µL aliquot of the resuspended sample was thoroughly mixed with 1 mL Coomassie brilliant blue G-250 reagent and incubated at room temperature for 10 min before measuring the absorbance at a wavelength of 595 nm. The protein concentration was determined according to a standard curve and the MCP content was calculated.

The NH_3_-N concentration was determined according to the method used by Zhang et al. [17]. and relevant national standards, which were optimized. Specifically, 1 mL rumen fluid was placed in a 2 mL sterile centrifuge tube and centrifuged at 12,000 ×*g* for 10 min at 4 °C (H2050R, Xiangyi Laboratory Instrument Development Co., Ltd., Hunan, China). The supernatant was passed through a 0.22 μm sterile filter membrane. A 1% phenol chromogenic solution containing 0.05% sodium nitroso ferricyanide (stored at 4 °C in darkness) and a fresh alkaline sodium hypochlorite solution were prepared. Standard working solutions were prepared using NH_4_Cl. After adding 1 mL sample or standard solution to a test tube, 2.5 mL phenol chromogenic solution and 2.0 mL alkaline sodium hypochlorite solution were added sequentially. After vortexing for 5 s, the mixture was incubated in a 37 °C-water bath for 30 min in darkness (covered with aluminum foil). After cooling to room temperature, the absorbance was measured at a wavelength of 650 nm, with distilled water used as a blank control. The NH_3_-N concentration (mg/100 mL) was calculated using a standard curve.

TVFA concentrations were determined as described by Li et al. [18] and in accordance with the national standard method. Briefly, 10 mL rumen fluid was centrifuged at 12,000 ×*g* for 10 min at 4 °C (H2050R, Xiangyi Laboratory Instrument Development Co., Ltd., Hunan, China). After collecting the supernatant, 25% phosphoric acid solution was added at a ratio of 4:1 (*v*/*v*). The resulting solution was thoroughly mixed by vortexing and then left standing at 4 °C for 30 min to precipitate proteins. The solution was centrifuged at 12,000 ×*g* for 10 min, after which the supernatant was passed through a 0.22 μm filter membrane and transferred to a gas chromatography vial for the subsequent analysis using a gas chromatograph (Agilent 7890B, Agilent Technologies, USA) equipped with a DB-FFAP capillary column (30 m × 0.32 mm × 0.25 μm) and FID detector. The parameters were set as follows: carrier gas, nitrogen at a flow rate of 400.0 mL/min; hydrogen flow rate, 40.0 mL/min; air flow rate, 400 mL/min; split ratio, 10:1; injection volume, 1.0 µL; injection port temperature, 200 °C; detector temperature, 280 °C. The column temperature program was as follows: initial temperature, 60 °C (held for 2 min); increased at 5 °C/min to 130 °C; increased at 15 °C/min to 190 °C (held for 3 min). 2-Ethylbutyric acid was used as the internal standard to prepare a mixed standard containing C2, C3, butyric acid (C4), isobutyric acid (iC4), and isovaleric acid (iC5). Each sample was injected three times and the average peak area was determined. Individual VFAs concentrations (mmol/L) and the TVFA concentration were calculated using an internal standard method.

### Analysis of the microbial community

The V3–V4 hypervariable region of the bacterial 16 S rRNA gene was amplified via a polymerase chain reaction (PCR) using total DNA extracted from PRC rumen microorganisms as the template. Microbial diversity profiling was performed using an Illumina NovaSeq 6000 sequencing platform. The universal primer pair 338 F (5′-ACTCCTACGGGAGGCAGCA-3′) and 806R (5′-GGACTACHVGGGTWTCTAAT-3′), which was used for the PCR amplification, was synthesized by Biomarker (BMK) Technologies (Beijing) Co., Ltd. The original data were optimized for analyses of ruminal bacterial 16 S rRNA gene alpha diversity, taxonomic differences in the ruminal microbial system, and ruminal bacterial 16 S rRNA gene species-level beta diversity.

### Construction and determination of the evaluation index system and weighting

To systematically evaluate the comprehensive effects of varying triticale silage substitution ratios for corn silage on the production performance, health metabolism, and rumen function of PRC, and to resolve the inconsistency of evaluation conclusions across individual indicators, this study employed the TOPSIS for multi-index decision analysis. This method computes the relative Euclidean distances between each experimental treatment group and both the “ideal best solution” and “ideal worst solution”; it then yields a closeness index that comprehensively reflects the relative merit of each treatment, ultimately enabling objective, quantitative ranking of the different substitution ratios.

The construction of the evaluation index system consists of five steps: (1) constructing a weighted normalized decision matrix; (2) constructing a weighted normalized decision matrix; (3) determining the positive ideal solution and negative ideal solution; (4) calculating the distance from each scheme to the ideal solution; (5) calculating the relative closeness and ranking.

#### Index selection and classification

From the dimensions of growth performance, digestive enzyme activity, serum biochemical, rumen fermentation, and bacterial community, a total of 17 key indicators with significant differences between groups (*P* < 0.05) or clear biological functions were selected to construct a comprehensive evaluation system. Based on their biological significance, all indicators were clearly classified into positive indicators (i.e., higher values are better, such as ADG, SOD activity) and negative indicators (i.e., lower values are better, such as NH_3_-N, CREA, UA).

#### Weight assignment

The subjective weighting method was adopted, and weights were directly assigned to each indicator based on the principle of “production performance as the core, with health and rumen ecology equally important” in the beef cattle fattening industry. When assigning weights, priority was given to core production performance indicators, while key parameters reflecting digestive metabolic efficiency and rumen microbial ecological balance were fully considered. The specific allocation scheme is detailed in Table 2.

**Table 2:** The comprehensive evaluation index system and weight assignment

Evaluation dimension	Evaluation mechanism	Items ^a^	Weight
Production performance	growth efficiency	ADG	0.18
	intake behaviour	TFI	0.07
		DMI	0.05
Health and metabolic status	digestive function	CT	0.06
		TS	0.06
		LS	0.04
	liver function	TBIL	0.04
		ALP	0.04
	renal function	CREA	0.03
		UA	0.04
	oxidative stress	SOD	0.05
	protein metabolism	NH_3_-N	0.06
Rumen function	key bacteria at phylum level	*Firmicutes*	0.06
		*Patescibacteria*	0.02
	key bacteria at genus level	*Prevotella*	0.07
		*Butyrivibrio*	0.06
		*Succiniciasticum*	0.03

^a^*ADG*, Average daily gain, *TFI*, Total feed intake, *DMI*, Dry mater intake, *CT*, Chymotrypsin, *TS*, Trypsin, *LS*, Lipase, *TBIL*, Total bilirubin, *ALP*, Alkaline phosphatase, *CREA*, Creatinine, UA, Uric acid, *SOD*, Superoxide dismutase, *NH*_3_*-N*, Ammonia nitrogen

#### TOPSIS model steps

##### Data preprocessing

Positive transformation: To unify the direction of indicators, all negative indicators were converted to positive ones. The transformation formula is: *x’*_*ij*_ *= max*(*x*_*j*_) *− x*_*ij*_. Where *x’*_*ij*_ is the original value of the *j*-th negative indicator in the *i*-th treatment group, and *max*(*x*_*j*_) is the maximum value of this indicator across all treatment groups. After transformation, all indicators become positive.

Normalization (standardization): To eliminate dimensional differences in the original data, vector normalization was applied to all positive-transformed data. The formula is: $\:{\mathrm{z}}_{\mathrm{ij}}\mathrm{=}\frac{{\mathrm{x}}_{\mathrm{ij}}^{{\prime\:}}}{\sqrt{{\sum\:}_{\mathrm{i}\mathrm{=1}}^{\mathrm{m}}\mathrm{(}{\mathrm{x}}_{\mathrm{ij}}^{{\prime\:}}{\mathrm{)}}^{\mathrm{2}}}}$. Where *m* is the number of treatment groups (*m* = 5), and *z*_*ij*_ is the normalized value.

##### Constructing the weighted normalized decision matrix

Multiply each element of the normalized decision matrix by the weight *w*_*j*_ corresponding to the index to obtain the weighted normalized matrix *V*: *v*_*ij*_ = *w*_*j*_⋅*z*_*ij*_. Where *v*_*ij*_ represents the weighted normalized value of the *i*-th scheme on the *j*-th index.

##### Determining the positive ideal solution and negative ideal solution

Positive ideal solution *V*^+^: Composed of the maximum values of each index in the weighted matrix *V*, representing the theoretically optimal scheme.

*V*^+^ = (*v*_1_^+^, *v*_2_^+^ …, *v*_*n*_^+^) =(*max*(*v*_1*j*_), *max*(*v*_2*j*_), …, *max*(*v*_*nj*_)).

Negative ideal solution *V*^−^: Composed of the minimum values of each index in the weighted matrix *V*, representing the theoretically worst scheme.

*V*^−^ = (*v*_1_^−^, *v*_2_^−^ …, *v*_*n*_^−^) =(*min*(*v*_1*j*_), *min*(*v*_2*j*_), …, *min*(*v*_*nj*_)).

##### Calculating the distance from each scheme to the ideal solution

Calculate the Euclidean distance from each treatment group (scheme) to the positive ideal solution and negative ideal solution.

The distance to the positive ideal solution: $\:{\mathrm{S}}_{\mathrm{i}}^{\mathrm{+}}\mathrm{=}\sqrt{{\sum\:}_{\mathrm{j}\mathrm{=1}}^{\mathrm{n}}\mathrm{(}{\mathrm{v}}_{\mathrm{ij}}\mathrm{-}{\mathrm{v}}_{\mathrm{j}}^{\mathrm{+}}{\mathrm{)}}^{\mathrm{2}}}$.

The distance to the negative ideal solution: $\:{\mathrm{S}}_{\mathrm{i}}^{\mathrm{-}}\mathrm{=}\sqrt{{\sum\:}_{\mathrm{j}\mathrm{=1}}^{\mathrm{n}}\mathrm{(}{\mathrm{v}}_{\mathrm{ij}}\mathrm{-}{\mathrm{v}}_{\mathrm{j}}^{\mathrm{-}}{\mathrm{)}}^{\mathrm{2}}}$. Where *n* is the total number of indicators (*n* = 17).

##### Calculating the relative closeness and ranking

Calculate the relative closeness *C*_*i*_ of each treatment group to the ideal solution: $\:{\mathrm{C}}_{\mathrm{i}}\mathrm{=}\frac{{\mathrm{S}}_{\mathrm{i}}^{\mathrm{-}}}{{\mathrm{S}}_{\mathrm{i}}^{\mathrm{+}}\mathrm{+}{\mathrm{S}}_{\mathrm{i}}^{\mathrm{-}}}$. The value of *C*_*i*_ ranges from 0 to 1. A larger *C*_*i*_ indicates a better comprehensive performance of the treatment scheme, which is closer to the ideal state. The treatment groups can be ranked in descending order of *C*_*i*_ values to obtain the comprehensive ranking.

### Statistical analysis

Data were preliminarily processed in Excel 2021, involving data entry, cleaning (outliers removed via Grubbs’ test with α = 0.05), and calculation of descriptive statistics (e.g., mean, standard deviation). Statistical analyses used IBM SPSS Statistics 24.0, where one-way ANOVA (preceded by Shapiro-Wilk normality and Levene’s variance homogeneity tests, both requiring *P* > 0.05) was applied with Tukey’s HSD post hoc comparisons (*P* < 0.05). Microbiological data were analyzed via BMKCloud (www.biocloud.net), including alpha diversity (Chao1, Shannon indices, Simpson index. etc.), beta diversity analysis (principal coordinate analysis (PCoA) based on weighted UniFrac distance), species annotation, and identification of differentially abundant taxa (Kruskal-Walli’s test, *P* < 0.05). Spearman’s correlation analysis was performed to assess relationships between main bacterial phylum (genus) (relative abundance > 1%) and rumen fermentation parameters were computed using individual samples (*n* = 30), with significance at *P* < 0.05 and strong correlations defined as |*r*| ≥ 0.5.

## Results

### Growth performance

As shown in Table 3, the substitution corn silage with triticale silage significantly affected ADG, TFI, DMI, TG, and FCR in PRC. ADG was significantly affected by the substitution level (*P* < 0.05). The ADG in the T0 group was significantly higher than that in T1, T2, T3, and T4 groups (*P* < 0.05), and overall, ADG decreased linearly with increasing triticale silage substitution. TFI was significantly affected by the substitution level (*P* < 0.01). The TFI in the T0 group was significantly higher than that in T2, T3, and T4 groups (*P* < 0.05), but no significant difference was observed between T0 and T1 groups (*P* > 0.05). In general, TFI decreased with increasing triticale silage substitution. Significant differences in DMI were observed among treatments (*P* < 0.01). The DMI in the T0 group was significantly higher than that in T2, T3, and T4 groups (*P* < 0.05), and DMI showed a decreasing trend as the proportion of triticale silage increased. TG was significantly affected by the substitution level (*P* < 0.05). The TG in the T0 group was significantly higher than that in T1, T2, T3, and T4 groups (*P* < 0.05), and TG gradually decreased with increasing substitution levels, showing a clear decreasing trend across treatments. FCR was significantly affected by the substitution level (*P* < 0.05). The FCR in the T0 group was significantly lower than that in T3 and T4 groups (*P* < 0.05), but no significant difference was found between T0 and T1 or T2 groups (*P* > 0.05). Overall, FCR increased with increasing triticale silage substitution.

**Table 3:** Effects of different proportions of triticale silage substitute for corn silage on production performance of PRC

Item ^a^	Treatment ^b^					SEM ^c^	*P*-value
	T0	T1	T2	T3	T4		
ADG (kg)	1.31a	1.15b	1.02bc	0.91c	0.85c	0.05	0.02
TFI (kg)	1530.00a	1480.50a	1410.20b	1340.40bc	1269.90c	38.50	< 0.001
DMI (kg)	17.00a	16.45ab	15.67bc	14.89c	14.11c	0.42	< 0.001
TG (kg)	117.90a	103.80b	91.50c	82.00cd	76.50d	5.20	0.02
FCR	12.98b	14.28ab	15.41ab	16.35a	16.60a	0.60	0.04

Each value represents the mean ± SEM of 6 replicates (*n* = 6)

^a^ADG, average daily gain; TFI, total feed intake; DMI, dry mater intake; TG, total gain; FCR, feed conversion ratio

^b^T0, 100% corn silage; T1, 30% triticale silage substitution; T2, 50% triticale silage substitution; T3, 70% triticale silage substitution; T4, 100% triticale silage

^c^SEM, standard error of the mean

Means in the same row with different superscript letters (a–c) differ significantly (*P* < 0.05)

### Enzyme activities in pancreatic juice and UUN in urine

Table 4 presents pancreatic juice enzyme activities and urine UUN concentrations. CT activities were significantly higher for the treatment groups (T1–T4) than for the group T0 (*P* < 0.01). TS activities were significantly higher for groups T1, T2, and T3 than for T0 (*P* < 0.01), but there was no significant difference between group T4 and T0 (*P* > 0.05). LS activity was significantly lower for group T4 than for T0 (*P* < 0.05), whereas the LS activities of groups T1, T2, and T3 did not differ significantly from that of T0 (*P* > 0.05). There were no significant differences in UUN concentrations between the treatment groups and T0 (*P* > 0.05).

**Table 4:** Effects of different proportions of triticale substitute for corn silage on enzyme activity in pancreatic juice and biochemical indexes in the urine of PRC

Items ^a^	Treatment ^b^					SEM ^c^	*P*-value
	T0	T1	T2	T3	T4		
CT (U/mg prot)	0.48d	0.63c	0.80b	0.94a	0.80b	0.04	< 0.001
TS (U/mg prot)	8.86b	10.59a	10.99a	11.10a	8.60b	0.34	< 0.001
LS (U/g prot)	15.79a	15.46a	14.97a	14.86a	11.17b	0.53	0.04
UUN (mmol/L)	43.04	40.26	40.41	34.77	33.48	1.42	0.14

Each value represents the mean ± SEM of 6 replicates (*n* = 6)

^a^CT, chymotrypsin; TS, trypsin; LS, lipase; UUN, urinary urea nitrogen

^b^T0, 100% corn silage; T1, 30% triticale silage substitution; T2, 50% triticale silage substitution; T3, 70% triticale silage substitution; T4, 100% triticale silage

^c^SEM, standard error of the mean

Means in the same row with different superscript letters (a–d) differ significantly (*P* < 0.05)

### Serum markers

As presented in Table 5, the TBIL concentration was significantly higher for group T4 than for T0 (*P* < 0.01). ALP activities were significantly higher for groups T3 and T4 than for T0 (*P* < 0.01). CREA concentrations were significantly higher for groups T3 and T4 than for T0 (*P* < 0.05). By contrast, UA concentrations were significantly lower for the treatment groups than for T0 (*P* < 0.05). SOD activities were significantly lower in groups T3 and T4 than in T0 (*P* < 0.05). Triticale silage substituted for corn silage did not influence ALT, AST, ALB, TP, DBIL, UREA, GLU, TRG, CHO, Ca, P, MDA, and GSH-Px concentrations or activities. However, as the proportion of triticale silage increased, ALT and AST activities increased, while the TRG content decreased.

**Table 5:** Effects of different proportions of silage triticale substitute for corn silage on serum markers in PRC

Items ^a^	Treatment ^b^					SEM ^c^	*P*-value
	T0	T1	T2	T3	T4		
ALT (U/L)	42.31	43.50	43.53	43.60	46.67	0.84	0.62
AST (U/L)	96.30	87.87	99.43	107.28	107.95	2.85	0.12
ALB (g/L)	39.56	38.03	40.90	41.09	41.24	0.63	0.50
TP (g/L)	57.28	59.41	59.25	57.60	54.32	1.08	0.64
TBIL (µmol/L)	12.59bc	10.85c	13.84b	14.19b	18.19a	0.73	< 0.01
DBIL (µmol/L)	5.25	4.90	5.30	5.99	6.75	0.35	0.52
ALP (U/L)	175.86b	176.66b	201.63ab	223.89a	225.58a	6.87	< 0.01
UREA (mmol/L)	2.40	2.78	2.77	2.81	3.82	0.19	0.16
CREA (µmol/L)	104.27b	103.79b	112.28ab	116.03a	122.15a	2.27	< 0.05
UA (µmol/L)	37.04a	28.48b	27.62b	28.99b	28.48b	1.21	< 0.05
GLU (mmol/L)	2.68	2.36	2.02	2.13	2.10	0.13	0.59
TRG (mmol/L)	0.16	0.15	0.14	0.14	0.14	0.01	0.99
CHO (mmol/L)	2.56	2.43	2.49	2.47	2.37	0.10	0.99
Ca (mmol/L)	2.26	2.17	2.16	2.20	2.23	0.01	0.06
P (mmol/L)	1.75	1.76	1.67	1.66	1.64	0.02	0.18
SOD (U/mL)	463.02a	484.49a	445.64ab	415.88bc	399.61c	9.61	< 0.01
MDA (mmol/mL)	3.31	3.30	3.69	3.86	3.88	0.22	0.88
GSH-Px (U/mL)	32.53	36.80	34.13	33.87	33.41	1.94	0.98

Each value represents the mean ± SEM of 6 replicates (*n* = 6)

^a^ALT, alanine aminotransferase; AST, aspartate aminotransferase; ALB, albumin; TP, total protein; TBIL, total bilirubin; DBIL, direct bilirubin; ALP, alkaline phosphatase; CREA, creatinine; UA, uric acid; GLU, glucose; TRG, triglycerides; CHO, cholesterol; Ca, calcium; P, phosphorus; SOD, superoxide dismutase; MDA, malondialdehyde, GSH-Px, glutathione peroxidase

^b^T0, 100% corn silage; T1, 30% triticale silage substitution; T2, 50% triticale silage substitution; T3, 70% triticale silage substitution; T4, 100% triticale silage

^c^SEM, standard error of the mean

Means in the same row with different superscript letters (a–c) differ significantly (*P* < 0.05)

### Fermentation parameters and volatile fatty acids

The rumen pH was within the normal range for the treatment groups and T0. The MCP content did not differ significantly between the treatment groups and CON (*P* > 0.05). By contrast, as the proportion of triticale silage increased, NH_3_-N concentration showed a downward trend (*P* < 0.01) (Table 6).

**Table 6:** Effects of different proportions of triticale substitute for corn silage on rumen fermentation parameters of PRC

Items ^a^	Treatment ^b^					SEM ^c^	*P*-value
	T0	T1	T2	T3	T4		
pH	6.17	6.22	6.15	6.14	6.09	0.11	0.99
MCP (mg/mL)	0.36	0.43	0.45	0.48	0.44	0.02	0.26
NH_3_-N (mg/100mL)	23.79a	19.62b	18.93c	18.00d	16.35e	0.67	< 0.01

Each value represents the mean ± SEM of 6 replicates (*n* = 6)

^a^ MCP, microbial crude protein; NH_3_-N, ammonia nitrogen

^b^ T0, 100% corn silage; T1, 30% triticale silage substitution; T2, 50% triticale silage substitution; T3, 70% triticale silage substitution; T4, 100% triticale silage

^c^ SEM, standard error of the mean

Means in the same row with different superscript letters (a–d) differ significantly (*P* < 0.01)

As indicated in Table 7, TVFA, C2, C3, C4, C5, iC4, iC5, C2/C3, C2/TVFA, and C4/TVFA did not differ significantly between the treatment groups and T0 (*P* > 0.05). However, with the increase in the proportion of triticale silage, the concentrations of TVFA, C2, C3, C4, C5, and iC5 showed a trend of first increasing and then decreasing.

**Table 7:** Effects of different proportions of triticale substitute for corn silage on rumen volatile fatty acids in PRC

Items (mmol/L) ^a^	Treatment ^b^					SEM ^c^	*P*-value
	T0	T1	T2	T3	T4		
TVFA	76.98	81.28	83.48	87.70	82.26	1.57	0.33
C2	54.55	57.80	58.65	59.12	59.62	0.93	0.50
C3	11.79	12.70	13.73	15.06	12.40	0.57	0.45
C4	7.69	7.76	7.99	9.85	7.06	0.38	0.18
C5	0.76	0.78	0.82	0.95	0.73	0.04	0.60
iC4	1.08	0.77	0.79	1.02	0.88	0.02	0.56
iC5	1.34	1.35	1.36	1.76	1.54	0.07	0.30
C2/C3	4.63	4.55	4.27	3.92	4.81	0.19	0.40
C2/TVFA	0.71	0.71	0.70	0.67	0.72	0.01	0.06
C4/TVFA	0.10	0.10	0.10	0.11	0.09	0.01	0.15

Each value represents the mean ± SEM of 6 replicates (*n* = 6)

^a^ TVFA, total volatile fatty acids; C2, acetic acid; C3, propionic acid; C4, butyric acid; C5, valeric acid; iC4, isobutyric acid; iC5, isovaleric acid

^b^ T0, 100% corn silage; T1, 30% triticale silage substitution; T2, 50% triticale silage substitution; T3, 70% triticale silage substitution; T4, 100% triticale silage

^c^ SEM, standard error of the mean

### Diversity of ruminal bacterial communities

**Fig. 1:**
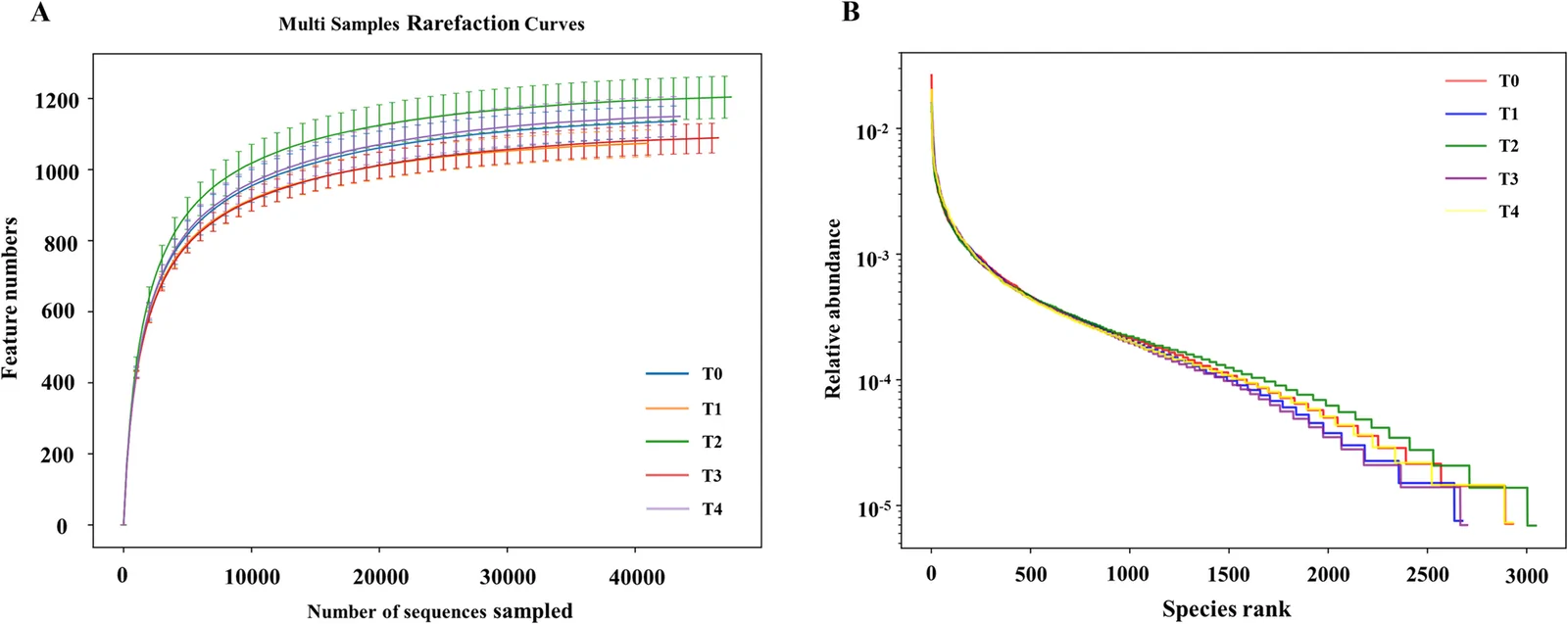
Dilution curve (**A**) and Rank Abundance curve (**B**)

The sample dilution curve plateaued when the number of sequences reached 30,000 (Fig. 1A). The plateauing of the sample dilution curve indicates that the sequencing depth is sufficient to cover most of the operational taxonomic units (OTUs) and the amount of data is within a reasonable range. If the number of sequences continues to increase, the number of OTUs will not increase significantly. The dilution curve is useful for determining whether the amount of sequencing data was sufficient to cover all groups. Additionally, it indirectly reflects species richness in the sample, which indicates whether the number of samples was reasonable. In this study, the sample dilution curve was relatively flat, indicating that the sequencing depth was sufficient, while also suggesting that the sample size was appropriate. In the rank abundance curve (Fig. 1B), the curve spanned a relatively large part of the horizontal axis and had a gradual downward trend along the vertical axis, indicating that sample species richness was high and species distribution was relatively uniform. According to the sample dilution curve (Fig. 1A) and the rank abundance curve (Fig. 1B), the sample size and sequencing depth were appropriate for the subsequent data analysis.

**Fig. 2:**
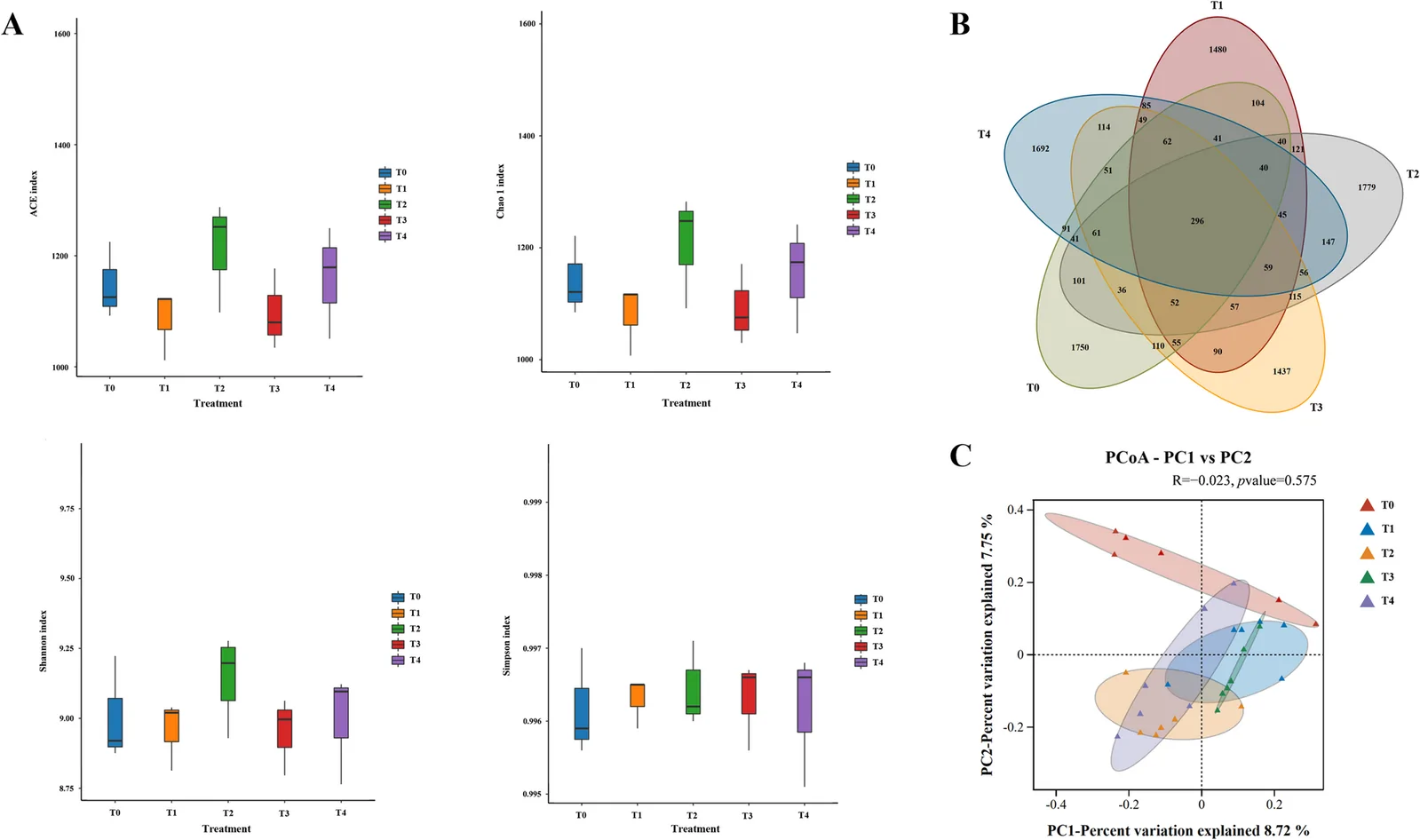
Effects of triticale silage substitution proportions on ruminal microbiota in PRC. (**A**) α-diversity, (**B**) Venn plots, and (**C**) β-diversity analysis

High-throughput sequencing coverage in each group was 99.96% (i.e., greater than 97%), indicating that sequencing depth was suitable for determining rumen bacterial types and community structures. A comparison with T0 indicated that ACE and Chao1 indices were higher for groups T2 and T4, but lower for groups T1 and T3 (Fig. 2A), but these differences were not significant (*P* > 0.05). Similar trends were observed for Simpson and Shannon indices (*P* > 0.05). Notably, with the increase in the proportion of triticale silage, the ruminal microbial abundance showed a trend of first increasing and then decreasing.

In this study, rumen content samples were collected from 30 PRC divided in five groups. On the basis of an Illumina HiSeq sequencing analysis, 1,199,739 paired reads were obtained from 30 rumen microbial samples. A total of 1,116,027 clean reads were retained after filtering raw reads for quality and splicing double-ended reads. At least 73,208 clean reads were obtained for each sample, with an average of 37,200 clean reads. To examine species diversity, we clustered the effective sequences into OTUs, with 97% consistency, and obtained a total of 10,257 OTUs. As presented in Fig. 2B, there were 296 core OTUs among the different groups. The number of OTUs unique to rumen microorganisms was highest and lowest in groups T2 (1,779) and T3 (1,437), respectively.

According to the results of a PCoA (Fig. 2C), there were significant differences in the microbial community composition among groups T1, T2, and T0. Although the groups were separated in the scatter plot, there was some overlap between groups T3 and T4. Thus, substituting corn silage with triticale silage may alter the microbial composition of PRC, but the substitution ratios used in this study did not result in significant differences between groups.

**Fig. 3:**
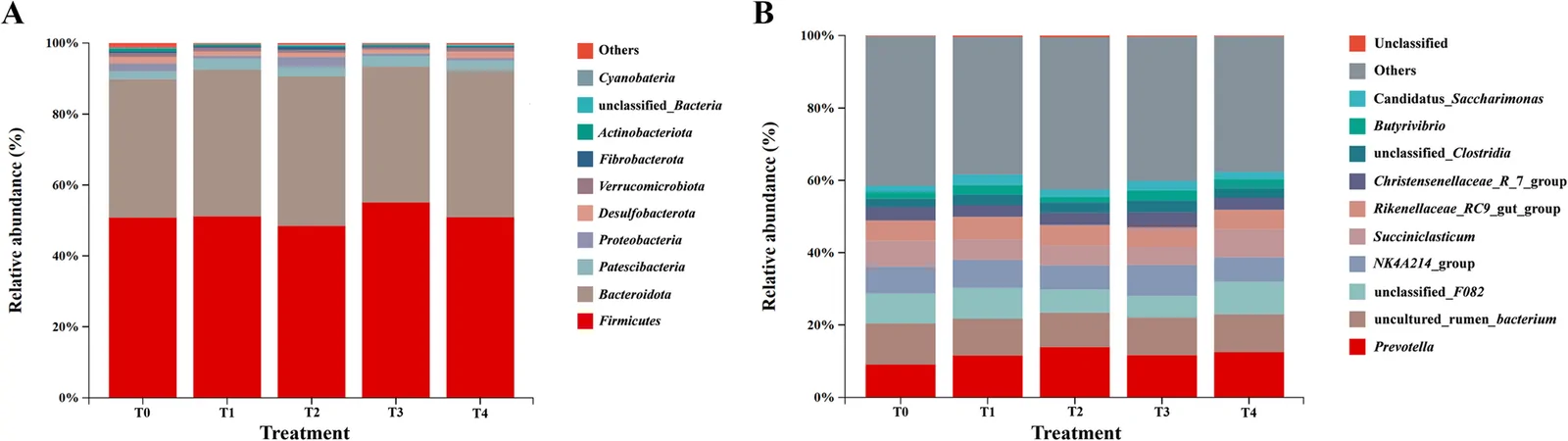
Relative abundance of bacterial composition. (**A**) At the phylum level and (**B**) at the genus level

Based on the microbial composition at the phylum level (Fig. 3A), *Firmicutes*, *Bacteroidota*, *Patescibacteria*, and *Proteobacteria* were the dominant rumen bacterial phyla across all treatments, collectively accounting for approximately 95% of the total bacterial population. The relative abundance of *Firmicutes* was higher in groups T1, T3, and T4 (51.14%, 55.08%, and 50.87%, respectively) than that in the T0 (50.81%). For *Bacteroidetes*, its relative abundance in groups T1, T2, and T4 (41.35%, 42.21%, and 41.38%, respectively) was significantly higher compared with the T0 (39.02%). *Patescibacteria* exhibited a higher relative abundance in groups T1, T2, T3, and T4 (3.10%, 2.61%, 2.93%, and 2.82%, respectively) than in the T0 (2.15%). Regarding *Proteobacteria*, its relative abundance in group T2 (2.78%) was higher than that in the T0 (2.26%).

At the genus level, the dominant rumen bacteria among treatments were as follows: *Prevotella*, uncultured_*rumen*_*bacterium*, unclassified_*F082*_group, and *NK4A214*_group. The proportion of rumen bacteria that were *Prevotella* species was higher for groups T1, T2, T3, and T4 (11.58%, 13.90%, 11.67%, and 12.53%, respectively) than for T0 (9.11%). By contrast, the proportion of rumen bacteria that were classified as uncultured_*rumen*_*bacterium* was lower for groups T1, T2, T3, and T4 (10.15%, 9.53%, 10.41%, and 10.45%, respectively) than for T0 (11.36%). Notably, the proportion of rumen bacteria belonging to unclassified_*F082*_group was higher for group T1 (8.54%) than for T0 (8.23%). The proportion of rumen bacteria that belonged to *NK4A214*_group was higher for groups T1 and T3 (7.71% and 8.50%, respectively) than for T0 (7.40%) (Fig. 3B).

**Fig. 4:**
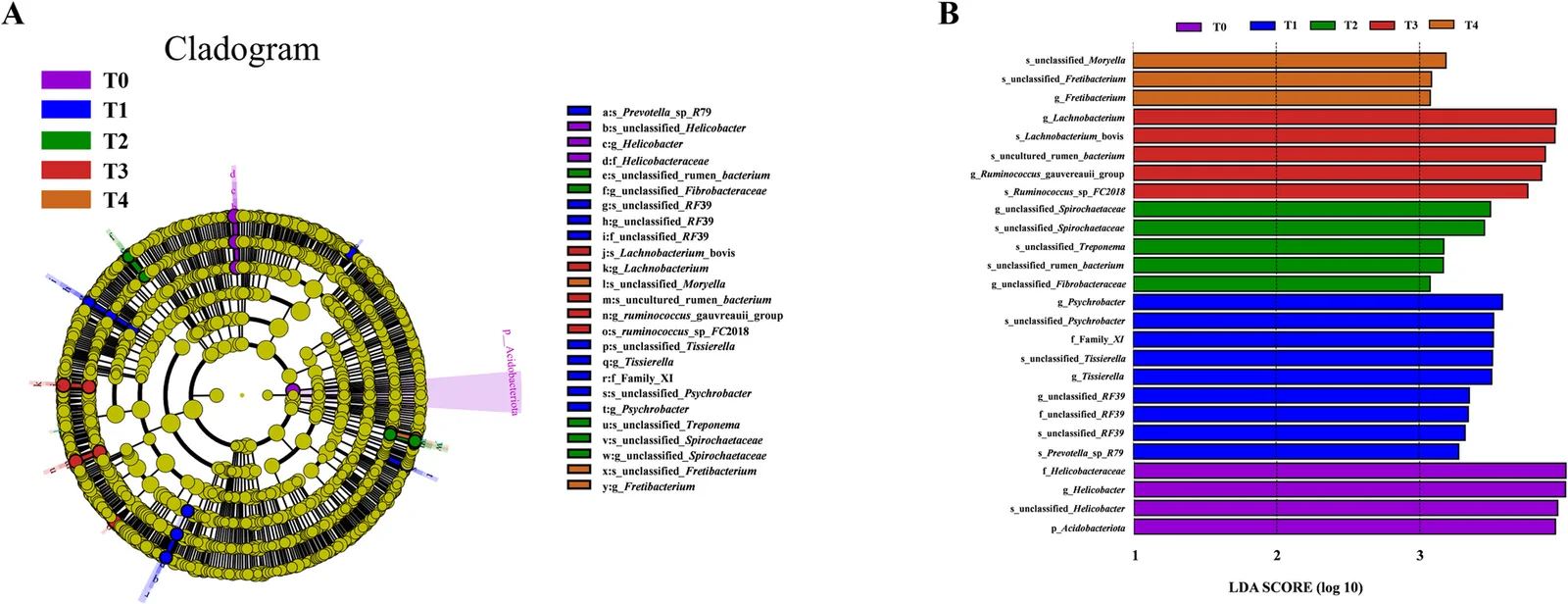
LEfSe analysis of microbial communities. (**A**) LEfSe taxonomic cladogram and (**B**) differential species score

On the basis of linear discriminant analysis (LDA) effect size, two or more groups were compared to identify microbial species that differed significantly between groups in terms of abundance. Additionally, specific biomarkers were screened. A total of 26 significantly different populations were identified (LDA > 3), of which four were enriched in the group T0, nine were enriched in group T1, five were enriched in groups T2 and T3, and three were enriched in group T4. The main species in the group T0 affecting the community structure were classified as *Helicobacteraceae*, *Helicobacter*, and *Acidobacteriota*. The main species in group T1 that influenced the community structure were classified as *Psychrobacter*, *Tissierella*, unclassified_*RF39*, and *Prevotella*_*sp*_*R79*. The main species in group T2 affecting the community structure were classified as unclassified_*Spirochaetaceae*, unclassified_*Treponema*, unclassified_*rumen*_*bacterium*, and unclassified_*Fibrobacteraceae*. The main species in group T3 influencing the community structure were classified as *Lachnobacterium*, uncultured_*rumen*_*bacterium*, and *Ruminococcus*. The main species in group T4 that affected the community structure were classified as unclassified_*Moryella* and *Fretibacterium* (Fig. 4).

### Correlations between microorganism compositions and rumen fermentation parameters

**Fig. 5:**
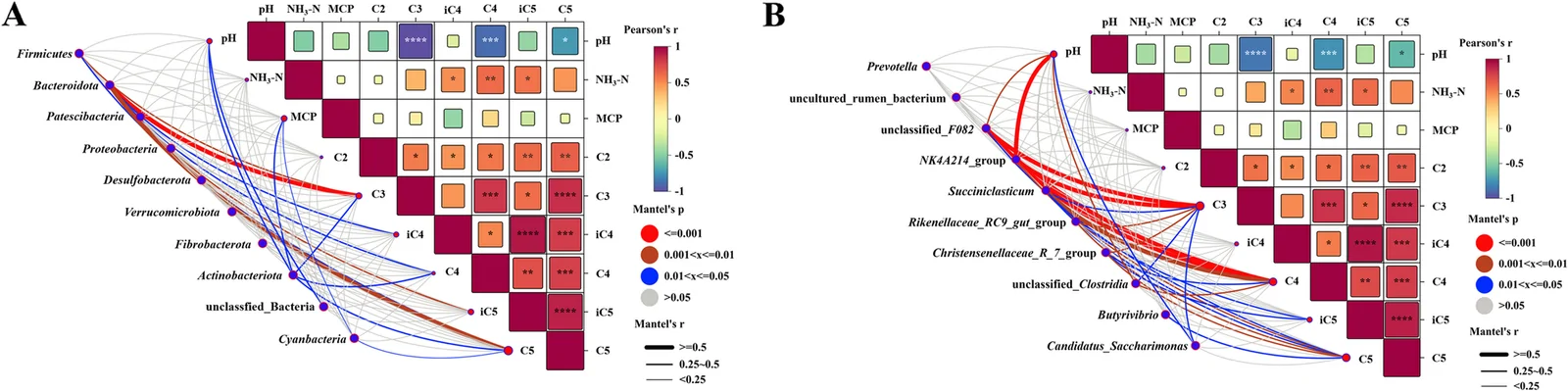
Correlation between the proportion of corn silage substituted with triticale silage and the bacterial community at the phylum level (**A**) and genus level (**B**) and rumen fermentation parameters. Triangles in the upper-right corner represent the correlation between rumen fermentation parameters, with different colors indicating Pearson correlation coefficients. Lines in the lower-left corner indicate the correlation between microorganism composition and rumen fermentation parameters. Microorganism composition parameters were obtained from partial Mantel tests. Line colors indicate positive (red) and negative (blue) correlations, whereas line thickness reflects the strength of the correlation (as determined by the correlation coefficient)

In terms of rumen fermentation parameters, pH was negatively correlated with MCP, C2, C3, C4, C5, iC4, iC5, and NH_3_-N (Fig. 5, upper-right corner). At the phylum level (Fig. 5A), pH was negatively correlated with *Actinobacteriota* (*r* = − 0.765, *P* < 0.001). C3 was negatively correlated with *Bacteroidota* (*r* = − 0.825, *P* < 0.001), but positively correlated with *Actinobacteriota* (*r* = 0.704, *P* < 0.01) and *Firmicutes* (*r* = 0.557, *P* < 0.05). C4 was positively correlated with *Actinobacteriota* (*r* = 0.593, *P* < 0.05). At the genus level (Fig. 5B), pH was negatively correlated with *NK4A214*_group (*r* = − 0.822, *P* < 0.001), *Christensenellaceae_R*_*7*_group (*r* = − 0.515, *P* < 0.05), and unclassified_*Clostridia* (*r* = − 0.770, *P* < 0.001), whereas C3 was positively correlated with *Candidatus*_*Saccharimonas* (*r* = 0.654, *P* < 0.01).

### Comprehensive evaluation results

The comprehensive evaluation results based on the TOPSIS model are presented in Table 8. group T1 (30% triticale silage substitution) achieved the highest composite score (*C*_*i*_ = 0.659) and ranked first among all treatments, indicating that this substitution level provided the optimal overall benefit. This suggests that a 30% substitution ratio optimally integrates the three core dimensions evaluated: growth performance, systemic metabolic health, and rumen function. By leveraging the nutritional advantages of triticale silage while avoiding the negative effects associated with higher substitution levels, this ratio-maintained growth performance comparable to the group T0, while significantly enhancing serum antioxidant capacity, reducing nitrogen metabolic load, and improving rumen fermentation efficiency and microbial community structure. Therefore, from a multi‑criteria decision‑making perspective, substituting 30% of corn silage with triticale silage represents the most balanced dietary strategy for fattening PRC, effectively supporting production efficiency, metabolic health, and rumen ecological stability.

**Table 8:** Comprehensive scores and rankings of each treatment based on TOPSIS

Treatment ^a^	S_i_^+^	S_i_^−^	Relative Closeness (Ci)	Rank
T0	0.0954	0.0767	0.446	3
T1	0.0581	0.1123	0.659	1
T2	0.0712	0.1045	0.595	2
T3	0.0826	0.0699	0.458	4
T4	0.1208	0.0511	0.297	5

^a^T0, 100% corn silage; T1, 30% triticale silage substitution; T2, 50% triticale silage substitution; T3, 70% triticale silage substitution; T4, 100% triticale silage

## Discussion

### Reasons for variation in cattle production performance

TG refers to the total increase in body weight over the entire trial period, and ADG is the corresponding daily increase, serving as a core indicator of growth rate. TG reflects the cumulative result of ADG and directly determines final meat production [19]. In this experiment, as the substitution level of triticale silage increased, ADG gradually decreased. The ADG values of groups T1, T2, T3, and T4 were significantly lower than those of T0, furthermore, TG exhibited a declining trend with increasing substitution levels. The primary reason for this phenomenon is the increase in substitution ratio, which caused a significant decreasing trend in ADG. This is mainly due to inherent differences in energy density and rumen fermentation patterns between the two silages: corn silage is rich in starch (ST) and other non‑fibrous carbohydrates, and its energy density is significantly higher than that of triticale silage. At the low substitution ratio (group T1), the diet could still provide sufficient ST, which is fermented in the rumen to produce a large amount of C3, meeting the animals’ glucose and energy requirements. Thus, ADG was maintained at a level comparable to that of the T0. However, when the substitution ratio reached or exceeded 50% (groups T2, T3, and T4), the ST content in the diet decreased sharply, leading to insufficient C3 production in the rumen and subsequent negative energy balance. Meanwhile, the relatively high content of anti‑nutritional factors (e.g., tannins (TA)) in triticale silage inhibits rumen microbial activity, reduces protein and fiber digestibility, and limits amino acid supply for muscle protein deposition. Ultimately, this resulted in a significant decrease in ADG, with the aforementioned negative effects becoming more pronounced as the substitution ratio increased [20, 21]. TG exhibited a decreasing trend as the proportion of triticale silage in the diet increased. This is because TG is the temporal accumulation of ADG, and the numerical differences in TG were partially buffered due to the fixed trial duration. Animals have a physiological regulatory mechanism that prioritizes resource allocation to maintenance metabolism rather than growth when facing nutritional limitation. However, the higher the substitution ratio, the more pronounced the negative impact of insufficient nutritional supply on growth efficiency, leading to a decreasing trend in TG [22].

TFI refers to the total amount of feed consumed by an individual or a group of livestock during the trial or feeding period (with leftover feed deducted), and it is the fundamental basis for calculating feed costs and FCR. DMI represents the average daily feed consumption, which can reflect the appetite status and feeding behavior of animals, and acts as an important reference for scientifically formulating nutritionally balanced diets [23]. In this study, TFI exhibited a decreasing trend. At low to moderate substitution levels (T1, T2, T3), although the palatability of triticale silage was slightly lower than that of corn silage, the overall dietary characteristics did not change fundamentally, and the rumen digestive burden remained within an adjustable range; thus, TFI did not differ significantly from T0. However, at complete substitution (T4), the cumulative effect of TA significantly reduced diet palatability. In addition, the relatively high content of physically effective neutral detergent fiber (peNDF) in triticale silage prolonged rumen retention time and enhanced satiety, ultimately leading to a significant decrease in TFI [24]. In this experiment, the decrease in DMI of PRC was driven by a combination of reduced diet palatability, enhanced rumen physical filling effects, and feedback regulation in response to negative energy balance [25].

FCR is defined as the amount of feed consumed per unit of weight gain, a key indicator for comprehensively evaluating breeding benefits and feed utilization efficiency; its value directly determines the economic benefits of production [26]. In the present study, as the proportion of triticale silage increased, FCR showed a decreasing trend. This is because with the increase in the substitution ratio of triticale silage, ADG and TFI decreased synchronously. Since FCR reflects the ratio relationship between feed consumption and weight gain, the magnitude of the synchronous decrease in ADG and TFI did not disrupt the original proportional balance, resulting in no significant difference in FCR among groups. However, inadequate nutrient supply in the high-substitution groups led to a latent reduction in growth efficiency, resulting in a downward trend in FCR [27].

### Reasons for changes in enzyme activities in pancreatic juice and UUN concentrations in urine

CT breaks proteins into smaller polypeptides, while TS, a pancreatic enzyme involved in intestinal digestion, decomposes proteins into absorbable oligopeptides. In the present study, the activities CT and TS first increased and then decreased with the increasing substitution proportion of triticale silage. The regulatory effect of triticale silage on pancreatic enzyme activities depends on the substitution ratio of corn silage. Specifically, at relatively low substitution ratios, the nutrient composition and moderate acidification of triticale silage enhance enzyme activities. However, when the proportion of triticale silage exceeds a critical threshold, enzyme activities decrease. This reduction is attributed to the accumulation of anti‑nutritional factors (e.g., TA and non‑starch polysaccharides), excessive pH decline, and microbial metabolic imbalance, moderate dietary complexity stimulates digestive function, whereas excessive fiber and secondary compounds impair enzymatic activity [28]. LS is a critical enzyme for lipid digestion that hydrolyzes fats into glycerol and fatty acids. In the current study, LS activity decreased with increasing substitution proportion of triticale silage. This phenomenon is mainly associated with the high fiber content and specific lipid structure of triticale silage: the former hinders effective contact between the enzyme and its substrate and alters the digestive tract environment, while the latter reduces enzymatic catalytic efficiency. Additionally, reduced dietary fat intake inhibits LS secretion via neuroendocrine feedback, and negative energy balance forces the body to redirect metabolic resources from energy‑consuming enzyme synthesis to life‑sustaining processes. Together, these factors mediate the metabolic regulation of digestion, reflecting how energy limitation prioritizes essential metabolic pathways [29].

UUN is primarily generated as a metabolic byproduct of dietary protein, which is hydrolyzed into amino acids in the digestive tract, converted into urea in the liver, and subsequently excreted via the kidneys. Mineral elements in silage also affect protein metabolic pathways and modulate UUN production [30]. In the present study, the UUN concentration in the urine of PRC generally decreased with increasing substitution levels of triticale silage, which comprehensively reflects the improved dietary nitrogen utilization efficiency. The high‑fiber content of triticale silage optimises the rumen fermentation environment by stimulating rumination and salivary secretion. Its specific fibre components synchronise NH_3_‑N release with microbial energy supply, while moderate levels of TA reduce ruminal protein degradability. Together, these factors promote the conversion of dietary nitrogen into microbial protein rather than urea, thereby decreasing UUN excretion and improving nitrogen retention [31].

### Factors driving changes in serum biomarkers

ALT, located in hepatocytoplasm, is involved in amino acid transport and metabolism, and plays a critical role in hepatic amino acid metabolism and gluconeogenesis. AST, widely distributed in animal tissues and cells, is primarily responsible for mediating transamination reactions between amino acids and keto acids. Elevated dietary fibre and reduced feed intake have been shown to impair liver function by increasing metabolic load and inducing nutrient imbalance [32]. In this study, the activities ALT and AST remained stable initially and then increased with increasing proportions of triticale silage. This phenomenon is mainly attributed to normal hepatic nutrient metabolism at appropriate substitution levels; however, excessive substitution reduces feed intake, increases hepatic burden, and causes hepatocellular damage. As the most abundant plasma protein in blood, ALB can maintain colloid osmotic pressure, while TP reflects systemic protein metabolism [33]. In the current study, TP and ALB levels remained stable at low substitution proportions but tended to decrease as the substitution proportion increased further. This suggests that moderate substitution sustains protein metabolic balance. However, as the substitution proportion increases, the elevated fiber content reduces the apparent digestibility of CP and decreases ruminal MCP synthesis efficiency, leading to an insufficient supply of available amino acids. Together with reduced intake of dietary available energy, these factors limit the availability of substrates and ATP required for hepatic protein synthesis, ultimately decreasing serum TP and ALB concentrations [34].

TBIL, including its DBIL and indirect bilirubin (IBIL), serves as a key indicator for assessing several critical physiological processes in animals, namely the production, uptake, conjugation, and excretion of bilirubin [35]. In the present study, serum TBIL and DBIL concentrations showed a trend of remaining stable first and then increasing with the elevated substitution proportion of triticale silage. At low substitution proportions, the dietary nutrition was balanced, and hepatic bilirubin metabolism remained normal. However, at high substitution levels, the elevated fibre content of triticale silage increases the metabolic load on the liver, impairs hepatocyte enzyme function, and interferes with bilirubin conjugation and excretion, ultimately leading to abnormal bile secretion and elevated serum TBIL and DBIL [36]. ALP is primarily derived from intrahepatic hepatobiliary duct epithelial cells. Under conditions of cholestasis or biliary stimulation, the synthesis and release of ALP are markedly enhanced, leading to a significant increase in its serum activity [37]. In this experiment, serum ALP activity increased with the elevated substitution proportion of triticale silage. At high substitution levels, the high fibre content and anti‑nutritional factors in triticale silage increase hepatic metabolic load, induce cholestasis, and stimulate ALP release from biliary epithelial cells. Impaired biliary excretion also promotes ALP reflux into the bloodstream. Moreover, differences in the calcium‑to‑phosphorus ratio between triticale and corn silage may disrupt biliary epithelial cell homeostasis, further exacerbating ALP release [38]. Collectively, these change primarily reflects the functional stress state of the hepatobiliary system.

In mammals, CHO serves as a precursor for the synthesis of various steroid hormones and bile acids. It also functions as a structural component of plasma lipoproteins, thereby influencing the transport and metabolism of low-density lipoprotein (LDL) and high-density lipoprotein (HDL). In the present study, serum CHO levels were observed to show a gradual decreasing trend with increasing substitution proportion of triticale silage. This change is primarily attributed to the synergistic effects of dietary fiber, phytosterols, and specific fatty acids in triticale silage. Dietary fiber and phytosterols inhibit intestinal cholesterol absorption by interfering with the enterohepatic circulation of bile acids; specific fatty acids can regulate hepatic cholesterol synthesis and LDL clearance pathways. Moreover, short‑chain fatty acids generated from fiber fermentation help maintain systemic cholesterol homeostasis by modulating the gut microbiota, ultimately resulting in reduced serum CHO concentrations [39].

SOD is a key enzyme in the animal antioxidant defense system. When animals are exposed to environmental stressors, significant amounts of reactive oxygen species (ROS) are generated, thereby triggering oxidative stress. Under oxidative stress conditions, free radical production exceeds the scavenging capacity of the antioxidant system, leading to cellular damage. GSH-Px is an important antioxidant enzyme in animals, which together with other antioxidant enzymes (e.g., SOD and CAT) constitutes the animal antioxidant defense system [40]. MDA is a well-established end product of lipid peroxidation. In animals under oxidative stress, reactive oxygen species attack polyunsaturated fatty acids in cell membranes, initiating a chain reaction of lipid peroxidation that generates MDA as a terminal byproduct, widely used as a biomarker for oxidative damage [41]. In the present study, serum SOD and GSH-Px activities showed a trend of remaining stable first and then decreasing, while MDA content exhibited an initial decrease followed by an increase with increasing substitution proportion of triticale silage. Moderate levels of dietary polyphenols and vitamins enhance antioxidant capacity, whereas excessive fiber and anti‑nutritional factors induce oxidative stress. At low substitution proportions, antioxidant components (e.g., polyphenols) in triticale silage synergistically enhanced the activities of antioxidant enzymes in the body, effectively inhibiting lipid peroxidation and reducing MDA levels. At high substitution proportions, however, increased dietary fiber load and antinutritional factors such as TA induced redox imbalance, which inhibited antioxidant enzyme activities, reduced free radical scavenging capacity, and exacerbated lipid peroxidation, ultimately leading to MDA accumulation [42].

### Fermentation parameters and factors influencing VFAs changes

The rumen pH is typically maintained between 5.5 and 7.5, a range conducive to the survival and proliferation of rumen microbes [43]. In this study, rumen pH remained within this normal range across all experimental groups, indicating that substituting corn silage with different proportions of triticale silage did not disrupt rumen homeostasis, thereby safeguarding rumen health. MCP synthesized by rumen microbes, dietary carbohydrates, and lipids are the primary and most direct nutrient sources for ruminants. The proliferation of rumen microbes can promote the conversion of non-protein nitrogen into ruminally available MCP, thereby significantly improving feed digestibility and nutrient utilization efficiency. In contrast, rumen MCP content and NH_3_-N concentration can reflect the decomposition and utilization efficiency of dietary nitrogen sources in the rumen [44]. MCP and NH_3_-N collectively form the core dynamic equilibrium system of ruminal nitrogen metabolism in ruminants. NH_3_-N serve as a key intermediate product of protein degradation and a direct nitrogen source for MCP synthesis, with its concentration reflecting the balance between dietary nitrogen degradation and microbial utilization. MCP, on the other hand, is a high-quality protein synthesized by microbes using NH_3_-N, meeting the majority of ruminants’ metabolic protein requirements. Its synthesis efficiency directly determines production performance. In this study, ruminal MCP content exhibited a trend of first increasing and then decreasing, while NH_3_-N concentration continued to decline with increasing substitution proportion of triticale silage. At appropriate substitution proportions, high-quality CP, minerals, and other nutrients in triticale silage promoted rumen microbial proliferation, enhanced MCP synthesis efficiency, and reduced NH_3_-N accumulation. At high substitution levels, however, increased dietary fibre load, altered fermentation patterns, and anti‑nutritional factors (e.g., TA) inhibited microbial activity, leading to impaired MCP synthesis. Although the continuous decline in NH_3_‑N indicates high microbial nitrogen capture efficiency, the failure to fully convert this nitrogen into MCP reveals a limitation in microbial metabolic efficiency under high‑substitution conditions [45].

VFAs are the core end products of carbohydrate metabolism in ruminants, and their production, absorption, and metabolism depend strongly on the structure, abundance, and function of the rumen microbial community. C2, the main precursor for fat synthesis, is directly used for fatty acid synthesis in mammary gland and adipose tissue. C3, a key substrate for hepatic gluconeogenesis, provides energy by being converted into glucose. C4 primarily supplies energy to the rumen epithelium, with a small portion participating in gluconeogenesis or ketogenesis. The composition and proportion of VFAs not only reflect ruminal fermentation characteristics but also affect the body’s energy allocation and metabolic health [46]. The C2 concentration and the C2/C3 ratio are closely associated with dietary fiber levels. A higher C2/C3 ratio reflects the dominance of structural carbohydrate (e.g., cellulose) fermentation, where enhanced activity of fiber-degrading bacteria promotes C2 production. Conversely, a lower ratio is linked to the fermentation of non-structural carbohydrates (e.g., ST), as propionate-producing bacteria utilize such substrates to increase C3 output. Therefore, the C2/C3 ratio can serve as a key indicator for assessing the shift in ruminal fermentation patterns from fiber degradation to ST digestion [47]. In this study, there was no significant difference in rumen TVFA content, component proportions among the groups. These results are primarily attributed to the “fermentation equivalence” between triticale silage and corn silage in carbohydrate composition: corn silage uses ST as its core fermentable substrate, while triticale silage is dominated by soluble sugars (SS) and high-quality fiber. Both provide highly fermentable substrates for the rumen, supporting similar metabolic pathways of rumen microbial fermentation. Additionally, the activity and community structure of ruminal fiber-degrading bacteria and acid-producing bacteria remained stable, with no significant fluctuations due to substrate substitution. Consequently, no statistically significant differences were observed in TVFA content, component proportions, and the C2/C3 ratio.

### Key factors affecting rumen microbial community diversity

The Alpha diversity indices are used to evaluate microbial community diversity within individual samples. The Chao1 and ACE indices reflect species richness, while the Simpson and Shannon indices assess species diversity and evenness. Based on these indices, differences in rumen microbial Alpha diversity of PRC among different treatment groups were compared. Studies have shown that rumen microbial community diversity is regulated by factors such as ruminant species, age, and diet composition. Within an appropriate range, an increase in dietary structural complexity helps expand microbial niches, thereby enhancing diversity. However, excessive complexity (e.g., increased fiber proportion) may inhibit the growth of some microbes, leading to a decrease in diversity [48]. This study demonstrated that although the Simpson and Shannon index did not show significant differences among the treatment groups, the ruminal microbial abundance exhibited a trend of first increasing and then decreasing with the increase in the proportion of triticale silage substitution. This phenomenon stemmed from the complementary effect of the two silages, corn silage provided abundant SS to promote the proliferation of lactic acid bacteria (LAB), while triticale silage supplied structural carbohydrates to maintain fermentation stability. At this ratio, the TVFA concentration increased moderately, and the buffering capacity of the feed kept the pH within a suitable range, creating an optimal environment for microbial growth. When the proportion of triticale silage is excessively high, excess SS causes excessive accumulation of TVFA, which lowers the ruminal pH below the optimal range. This, in turn, inhibits the growth of certain rumen microorganisms, leading to a decrease in their abundance [49].

The fermentation characteristics of silage and the succession of its microbial community significantly influence the composition of the rumen microbiota. Analysis at the phylum level revealed that *Firmicutes* and *Bacteroidetes* were the dominant bacterial groups in the rumen of PRC, followed by *Patescibacteria* and *Proteobacteria*. In the present study, at the phylum level, the ruminal bacterial community in PRC was primarily composed of *Firmicutes* and *Bacteroidetes*, with *Patescibacteria* and *Proteobacteria* present as secondary groups. This distribution reflects the selective pressures imposed by host physiological needs, microbial functional adaptation, and silage fermentation characteristics. *Firmicutes* efficiently degrade carbohydrates from silage and tolerate the slightly acidic ruminal environment, while *Bacteroidetes* play a dominant role in polysaccharide and protein metabolism. Their metabolic synergy forms the core functional group for nutrient transformation in the rumen, highlighting its role as an efficient fiber fermentation system. In contrast, *Patescibacteria* depend on metabolites from dominant bacteria for survival, and *Proteobacteria* serve only auxiliary functions in nutrient metabolism. As secondary taxa, their presence enhances community complexity and functional redundancy, collectively maintaining the stability and functional efficiency of the ruminal microbial ecosystem. *Prevotella* was identified as a core functional genus within the rumen microbiome, playing a key role in degrading hemicellulose and non-structural carbohydrates, as well as protein metabolism (e.g., polypeptide hydrolysis and amino acid conversion). Furthermore, this genus engages in synergistic interactions with fibrolytic bacteria (e.g., *Ruminococcus*) and acid-producing bacteria via metabolic complementation and cross-feeding mechanisms. These interactions regulate the proportional production of VFA and maintain rumen pH homeostasis, thereby sustaining the stability of fermentation efficiency and overall system function [50]. In the present study, at the genus level, the rumen bacteria of PRC formed a community structure where *Prevotella* and uncultured_*rumen*_*bacteria* were the dominant taxa, followed by unclassified_*F082*_group and *NK4A214*_group. This distribution pattern is the combined outcome of function-driven niche differentiation, limitations in rumen microbial culture techniques, advances in taxonomic research, and the selective effect of silage. In addition, the increased substitution ratio of triticale silage in the diet further promoted the enrichment of *Prevotella*, as its high content of structural carbohydrates serves as specific substrates for this genus. These four taxa form an efficient and stable fermentation network through functional complementarity, collectively ensuring the rumen’s continuous capacity to convert complex plant-based feed and maintaining the stability and functional integrity of the rumen microecosystem [51].

### Mechanism underlying the correlation between microorganisms and rumen fermentation parameters

Silage characteristics, rumen microorganisms, and fermentation parameters form a “substrate-microbiota-host” tripartite regulatory system. Silage directly shapes the rumen microbial community structure through its nutritional components and fermentation quality, driving the microbiota to influence key fermentation parameters (e.g., VFAs profiles and NH_3_-N conversion) via synergistic metabolism. Meanwhile, the host exerts feedback regulation on the microbiota through rumen environmental homeostasis. Optimizing silage fermentation technology can systematically improve rumen fermentation patterns, enhance microbial protein synthesis and feed conversion efficiency, and ultimately achieve synergistic improvements in ruminants’ productive performance and health status. In this study, pH was negatively correlated with MCP, C2, C3, C4, C5, iC4, iC5, and NH_3_-N. This observation aligns with the findings of He et al. [52], who reported a significant post-feeding pH decrease at 4 h, primarily attributed to VFA accumulation from enhanced microbial fermentation. Furthermore, the addition of TA significantly reduced the NH_3_-N concentration by inhibiting protein degradation without markedly affecting pH, indicating their differential regulatory effects on nitrogen metabolism and fermentation parameters [53]. Rumen microbial activity and abundance are modulated by pH. At optimal pH, microbial protein synthesis and MCP contents may increase, whereas suboptimal pH may have the opposite effects. Chen et al. [54] demonstrated that under specific dietary conditions, MCP is negatively correlated with the C2/C3 ratio and positively correlated with C3 concentration, which is consistent with the results of the present study. Zheng et al. [55] reported that ruminal pH is significantly negatively correlated with the relative abundance of *Actinobacteriota*, implying that pH changes may influence *Actinobacteriota* abundance and diversity under certain conditions. In the current study, C3 was negatively correlated with *Bacteroidota* but positively correlated with *Actinobacteriota* and *Firmicutes*. *Bacteroidota* predominantly mediates cellulose degradation and metabolism, which mainly produces VFAs (including C2 and C4); this metabolic pathway competes with C3 synthesis for substrates, so increased *Bacteroidota* abundance tends to reduce C3 concentration. When a relatively high proportion of corn silage was substituted with triticale silage, the dietary abundance of structural carbohydrates (e.g., NDF) increased significantly, promoting *Bacteroidota*-dominated fiber degradation and C2 production to inhibit C3 synthesis. In contrast, *Firmicutes* and *Actinobacteriota* directly utilize soluble carbohydrates for efficient propionate synthesis through the succinate-acrylate pathway, and their metabolic intermediates such as lactic acid (La) further stimulate the activity of propionate-producing bacteria. The elevated dietary NDF content also promoted *Bacteroidota* proliferation, while residual water-soluble carbohydrates (WSC) and soluble proteins (SP) sustained *Firmicutes* and *Actinobacteriota* metabolism; simultaneously, increased ruminal pH and reduced hydrogen partial pressure optimized conditions for propionate-producing bacteria, leading to significant C3 synthesis enhancement [56]. In the present study, C4 was significantly positively correlated with *Actinobacteriota*, suggesting that C4, a key product of energy metabolism, plays a critical role in genomic activities governing rumen nutrient utilization. Bacterial species within *Actinobacteriota*, particularly those of the *Clostridium* and *Eubacterium* genera, are critical for C4 production [57]. In the current study, ruminal pH was negatively correlated with *NK4A214*_group, *Christensenellaceae*_*R*_*7*_group, and unclassified_*Clostridia*. He et al. [58] reported that the relative abundance of *NK4A214*_group was significantly increased in the hot water treatment group and positively correlated with ruminal C3 levels. Given that C3 elevation is typically accompanied by ruminal pH reduction, a potential negative correlation between *NK4A214*_group abundance and ruminal pH is suggested. Additionally, *Christensenellaceae_R_7_*group is positively correlated with ruminal C3 concentration, lipid metabolism, and feed efficiency, while unclassified_*Clostridia* participates in ruminal carbohydrate degradation, their elevated abundance may be closely associated with ruminal pH reduction [59].

### Strengths, limitations and future perspectives

The major strength of this study lies in the integration of growth performance, serum biochemical indices, rumen fermentation parameters, and rumen microbial community into a TOPSIS-based multidimensional evaluation framework. This approach enabled the identification of a 30% substitution ratio of triticale silage for corn silage as optimal for PRC, and further revealed the underlying mechanisms, including enrichment of beneficial microbial taxa, improved nitrogen metabolism, and coordinated enhancement of multi-system functions. However, several limitations should be acknowledged. The study was conducted using a single breed at a single location, with relatively short silage fermentation and feeding durations. In addition, the mechanistic interpretation remains limited, as it lacks causal validation through multi-omics approaches and fine-scale resolution of microbial functional components. In light of these limitations, future research should focus on integrative multi-omics analyses, long-term and multi-site validation trials across the full production cycle, environmental sustainability assessments, targeted regulation of functional microbiota, and the development of precision nutrition models. These efforts will further facilitate the efficient utilization of triticale silage in regional ruminant production systems.

## Conclusions

Based on TOPSIS analysis integrating growth performance, serum biochemistry, pancreatic enzyme activities, rumen fermentation, and the microbial community, a 30% substitution ratio was identified as optimal. At this level, production performance remained comparable to T0, while multi-system functions were synergistically improved: enhanced pancreatic enzyme activities (CT and TS) promoted protein digestion; increased serum ALP activity, improved TRG metabolism, and reduced UUN indicated more efficient nitrogen utilization; decreased ruminal NH_3_-N together with increased MCP, DMI and TVFA reflected enhanced rumen fermentation; and increased relative abundances of beneficial bacteria (*Firmicutes*, *Bacteroidota*, and *Prevotella*), along with suppression of potential pathogens, supported a balanced rumen microbial ecosystem. Therefore, a 30% substitution of corn silage with triticale silage is recommended for PRC to achieve integrated benefits across digestive, metabolic, and microbial dimensions without compromising growth performance, providing a scientific basis for the rational application of triticale silage in ruminant production.

## Data Availability

The sequence data that support the findings of this study have been deposited in the NCBI’s Sequence Read Archive under Bio-Project accession number PRJNA1397596. (https://dataview.ncbi.nlm.nih.gov/object/PRJNA1397596).
